# Activation of Nrf2 Pathway Contributes to Neuroprotection by the Dietary Flavonoid Tiliroside

**DOI:** 10.1007/s12035-018-0975-2

**Published:** 2018-03-05

**Authors:** Ravikanth Velagapudi, Abdelmeneim El-Bakoush, Olumayokun A. Olajide

**Affiliations:** 1 Department of Pharmacy, School of Applied Sciences, University of Huddersfield, Queensgate, Huddersfield, HD1 3DH UK; 2 Present Address: Laboratory of Neurobiology, National Institute of Environmental Health Sciences, National Institutes of Health, Research Triangle Park, NC USA

**Keywords:** Tiliroside, Neuroinflammation, Neuroprotection, Nrf2, SIRT1, BV2 microglia, HT22 hippocampal neurons

## Abstract

Hyperactivated microglia plays a key role in regulating neuroinflammatory responses which cause damage to neurons. In recent years, substantial attention has been paid in identifying new strategies to abrogate neuroinflammation. Tiliroside, a natural dietary glycosidic flavonoid, is known to inhibit neuroinflammation. This study was aimed at investigating the molecular mechanisms involved in the inhibition of neuroinflammation and neurotoxicity by tiliroside. The effects of tiliroside on Nrf2 and SIRT1 activities in BV2 microglia and HT22 hippocampal neurons were investigated using immunoblotting and DNA binding assays. The roles of Nrf2 and SIRT1 in the anti-inflammatory activity of tiliroside were further investigated using RNA interference experiments. HT22 neuronal viability was determined by XTT, calcium influx, DNA fragmentation assays. The effect of tiliroside on MAP2 protein expression in HT22 neurons was investigated using western blotting and immunofluorescence. We also studied the impact of tiliroside on DNA fragmentation and ROS generation in APPSwe-transfected 3D neuronal stem cells. Results show that tiliroside increased protein levels of Nrf2, HO-1 and NQO1, indicating an activation of the Nrf2 protective mechanisms in the microglia. Furthermore, transfection of BV2 cells with Nrf2 siRNA resulted in the loss of anti-inflammatory activity by tiliroside. Tiliroside reduced protein levels of acetylated-NF-κB-p65, and increased SIRT1 in LPS/IFNγ-activated BV2 microglia. RNAi experiments revealed that inhibition of neuroinflammation by tiliroside was not affected by silencing SIRT1 gene. Results of neurotoxicity experiments revealed that neuroinflammation-induced toxicity, DNA fragmentation, ROS generation and calcium accumulation in HT22 neurons were significantly reduced by tiliroside treatment. In addition, the compound also protected differentiated human neural progenitor cells by blocking ROS generation and DNA fragmentation. Overall, this study has established that tiliroside protected BV2 microglia from LPS/IFNγ-induced neuroinflammation and HT22 neuronal toxicity by targeting Nrf2 antioxidant mechanisms. The compound also produced inhibition of NF-κB acetylation through activation of SIRT1, as well as increasing SIRT1 activity in mouse hippocampal neurons. Results from this study have further established the mechanisms involved in the anti-neuroinflammatory and neuroprotective activities of tiliroside.

**Electronic supplementary material:**

The online version of this article (10.1007/s12035-018-0975-2) contains supplementary material, which is available to authorized users.

## Introduction

Several studies indicate that neuroinflammation is a double-edged sword that executes both beneficial and detrimental effects on adjacent neurons in the brain [1]. This execution depends on the functional phenotype of the microglia, which ranges from pro-inflammatory M1 phenotype to immunosuppressive M2 phenotype. Hyperactive microglia that releases pro-inflammatory cytokines such as tumour necrosis factor-alpha (TNFα), interleukin 1 beta (IL-1β), interleukin 6 (IL-6), as well as superoxide, reactive oxygen species (ROS), nitric oxide (NO) and other critical pathways are termed as M1 microglia. On the other hand, M2-microglia have been shown to inhibit inflammatory responses by releasing anti-inflammatory cytokines such as IL-4, IL-13, IL-10 and transforming growth factor beta (TGF-β) [2, 3]. Studies have also shown that M2-microglia could suppress the production of pro-inflammatory cytokines, neurotoxic factors and reduce NO release, which collectively blocks lipopolysaccharide (LPS)-induced neuroinflammation [4, 5]. New findings indicate that the activation of intracellular anti-inflammatory mechanisms such as nuclear factor erythroid 2 related factor 2 (Nrf2) signalling could be a popular strategy to prevent inflammation-mediated neuronal toxicity. Recent studies revealed that Nrf2-deficient mice showed increased levels of neuroinflammatory M1 markers (cyclooxygenase 2 [COX-2], inducible nitric oxide synthase [iNOS], IL-6 and TNF) and reduced levels of M2 markers (Arginase 1 [ARG1] and IL-4) in response to neurotoxins [6, 7]. Also, other studies have suggested that nuclear factor kappa-light-chain-enhancer of activated B cells (NF-κB) is considered as a master regulator of M1 phenotype, while Nrf2 may be a regulator of the M2 phenotype [8].

Nrf2 is also known as a critical regulator of endogenous inducible defence systems in the brain and is actively produced by microglia in response to oxidative stress. Several studies have suggested the relevance of these antioxidant proteins in the immunomodulation of microglia [9]. Notably, heme oxygenase (HO-1) and NAD(P)H quinone dehydrogenase 1 (NQO1) are critical cytoprotective mediators in the cellular response to nitrosative and oxidative stress during neuroinflammation. Its activation has been shown to suppress LPS-induced inflammation in mouse peritoneal macrophages and microglial cells in the brain [10, 11]. From this perspective, targeting Nrf2/HO-1/NQO1-antioxidant axis with suitable pharmacologically active compounds may help to reduce neuroinflammation and its associated neurodegeneration.

In activated microglia, NF-κB signalling should undergo several post-translational modifications to regulate the transcription of pro-inflammatory genes. One such modification is reversible acetylation of NF-κB-p65 subunit. Studies have shown that sirtuins negatively regulate NF-κB signalling via deacetylation of Lys310 residue of RelA/p65 subunit [12, 13]. Sirtuins belong to class III histone deacetylases (HDACs), which plays a significant role in regulating ageing, inflammatory diseases and senescence by targeting histones, coregulatory and inflammatory transcription factors like NF-κB, p53 and Nrf2 in brain cells [14, 15]. Among the seven sirtuins, Sirtuin 1 (SIRT1) has been reported to be involved in promoting longevity in various species. Thus, activation of SIRT1 inhibits NF-κB signalling by promoting deacetylation of the p65 subunit. Also, reports have shown that inhibition of SIRT1 protein expression by LPS in endothelial cells significantly increased the expression of acetylated-NF-κB-p65 and further enhanced NF-κB transcriptional activity [16]. Moreover, microglia treated with LPS has significantly increased the production of inflammatory cytokines like IL-1β and TNFα, coinciding with the inhibition of SIRT1, suggesting that SIRT1 is involved in regulating pro-inflammatory cytokines [17, 18]. Consequently, there is an urgent need to investigate compounds that activate SIRT1 expression in neuroinflammation-driven neurological diseases.

Previously, we have reported inhibition of neuroinflammation by tiliroside in lipopolysaccharide and interferon gamma (LPS/IFNγ)-induced BV2 microglia [19]. However, nothing is known about the roles of Nrf2/ARE and SIRT1 signalling mechanisms in the anti-inflammatory activity of the compound. Also, very little is known about the potential neuroprotective activity of tiliroside. Therefore, we investigated Nrf2/ARE and SIRT1 activation by tiliroside in BV2 microglia and determined their roles in the anti-inflammatory activity of the compound. We also investigated neuroprotective actions of the compound in cultured mouse hippocampal neurons and immortalised human 3D neural stem cells.

## Materials and Methods

### BV2 Murine Microglia Cell Culture

BV2 microglia were obtained from Interlab Cell Line Collection, Banca Biologicae Cell Factory, Italy, and were cultured using Roswell Park Memorial Institute medium 1640 (RPMI) supplemented with 10% foetal bovine serum (FBS) (Sigma), 2 mM L-glutamine (Sigma), 100 mM sodium pyruvate (Sigma), 100 U/ml penicillin and 100 mg/ml streptomycin (Sigma) in a 5% CO_2_ incubator at 37 °C. Cells were passaged twice a week in 75 cm^2^ filter-capped vented flasks. Once confluent, flasks were washed with phosphate-buffered saline (PBS) and trypsinised with 2 ml of 0.25% trypsin-EDTA solution. Cells were then seeded out at a concentration of 2 × 10^5^ cells/ml in various cell culture plates.

### HT22 Mouse Hippocampal Neurons

HT22 neuronal cells were a kind gift from Dr. Jeff Davis. These cells were cultured in DMEM supplemented with 10% FBS, 100 mM sodium pyruvate (Sigma), 100 U/ml penicillin and 100 mg/ml streptomycin in a 5% CO_2_ incubator at 37 °C. Cells were cultured in 75 cm^2^ flasks. Confluent monolayers were washed with PBS and trypsinised with 2 ml of 0.25% trypsin-EDTA solution. Later, cells were seeded at a concentration of 2 × 10^5^ cells/ml in various cell culture plates.

### 3D Human Neural Progenitor (ReNcell VM) Cell Culture

Immortalised neural progenitor cells (ReNcell VM cells) were acquired commercially from Millipore (Hertfordshire, UK). Cells were maintained on laminin-coated 75 cm^3^ culture flask (Sarstedt, UK) in ReNcell NSC maintenance medium (Millipore, UK) containing freshly prepared epidermal growth factor (20 ng/ml; Gibco) and fibroblast growth factor-2 (20 ng/ml; Gibco). At 80% confluence, cells were detached with 4 ml accutase (Millipore, UK). Cell suspension was then centrifuged at 300×*g* for 3 min, and re-suspended in ReNcell NSC maintenance medium containing fresh EGF and FGF-2, and incubated at 37 °C in 5% CO_2_. To determine whether undifferentiated ReNcell VM cells express βIII-tubulin, a marker for human neural progenitor cells, an immunocytochemistry experiment was carried out using Alexa Fluor 488 anti-βIII-tubulin antibody (Biolegend, UK).

### Drugs and Treatment

Tiliroside was purchased from Sigma and prepared in DMSO. Primary stock of 100 mM of the compound was made and stored in small aliquots at − 80 °C. A working stock of 10 mM was prepared from aliquots of the original stock. The combination of LPS (100 ng/ml) and IFNγ (5 ng/ml) was used to stimulate BV2 microglia in all neuroinflammation-associated experiments. LPS was derived from *Salmonella enterica* serotype Typhimurium SL118, purchased from Sigma. IFNγ was derived from *Escherichia coli*, obtained from R & D systems. BV2 cells were seeded at a density of 2 × 10^5^ cells/ml in a six-well plate and incubated at 37 °C. Once confluent, cells were pre-treated with tiliroside (2–6 μM) for 30 min and stimulated with LPS (100 ng/ml)/IFNγ (5 ng/ml) for 24 h. Stimulation was terminated by removing supernatants (conditioned medium) from the cells and centrifuged at 2500 rpm for 5 min to remove cellular debris, aliquoted and then stored at − 80 °C.

### Antioxidant Responsive Element Reporter Assay

Cultured BV2 cells were harvested and seeded at a density of 4 × 10^5^ cells/ml in a solid white 96-well plate using Opti-MEM® (modified Eagle’s Minimum Essential Media) (Invitrogen) mixed with 5% FBS. Thereafter, pGL4.37 [luc2P/ARE/Hygro] vector (1 ng DNA/μl) was mixed with Fugene 6 transfection reagent and added to the cells followed by 16-h incubation at 37 °C. Thereafter, media was changed to Opti-MEM® (without 5% FBS) and incubated for a further 8 h. Transfected cells were treated with tiliroside (2–6 μM) for 24 h to investigate antioxidant responsive element (ARE)-associated gene expression. At the end of the stimulation, 100 μl of luciferase assay buffer containing luminescence substrate was added to each well and luminescence was read with FLUOstar OPTI microplate reader (BMG LABTECH).

### ARE DNA Binding Assay

To investigate DNA binding of Nrf2, BV2 microglia were treated with tiliroside (2–6 μM) for 24 h and nuclear extracts were prepared using EpiSeeker Nuclear Extraction Kit (Abcam). Then, 20 μg of nuclear lysates was added to 96-well plates on which oligonucleotide containing the ARE consensus binding site (5′ GTCACAGTGACTCAGCAGAATCTG-3′) has been immobilised. Thereafter, 30 μl of complete binding buffer was added to each well and incubated for 60 min at room temperature while shaking. The contents of the plate were discarded and the plate washed three times with 200 μl/well of wash buffer, followed by addition of 100 μl of Nrf2 antibody (1:1000). The plate was covered and incubated for further 60 min at room temperature while shaking, followed by three times washing. Then, 100 μl of HRP-conjugated antibody (1:1000) was added and incubated for further 60 min at room temperature followed by washing. Finally, 100 μl of developing solution was added and incubated in the dark for 15 min and the reaction was terminated by adding 100 μl of stop solution. Absorbance was read on a Tecan F50 microplate reader at 450 nm.

### RNA Interference

RNAi was used to determine whether Nrf2 activity plays a role in the inhibition of neuroinflammation by tiliroside. Nrf2 gene was silenced in BV2 microglia using Nrf2 siRNA (Santa Cruz Biotechnology). BV2 cells were cultured and seeded in a six-well plate at a density of 2 × 10^5^ cells/ml using antibiotic-free (penicillin and streptomycin) RPMI 1640 growth medium and incubated at 37 °C in a 5% CO_2_ incubator until 50% confluent. In one tube, 2 μl of Nrf2 siRNA duplex (Santa Cruz Biotechnology) was diluted into 100 μl of siRNA transfection medium (Santa Cruz Biotechnology). In a second tube, 2 μl of transfection reagent (Santa Cruz Biotechnology) was diluted into 100 μl of siRNA transfection medium. The contents of both tubes were gently mixed to and incubated for 45 min at room temperature. Next, 200 μl of the Nrf2 siRNA transfection cocktail was added to BV2 cells and further incubated for 6 h at 37 °C. Control BV2 microglia were transfected with control siRNA. Following transfection, media was changed to RPMI 1640 growth media and incubated for a further 18 h at 37 °C. Effects of tiliroside (6 μM) on nitrite, PGE_2_, TNFα and IL-6 production in LPS (100 ng/ml)/IFNγ (5 ng/ml)-stimulated control siRNA and Nrf2-siRNA-transfected BV2 cells were then determined. Also, iNOS and COX2 protein levels were evaluated using western blot, while NF-κB DNA binding was evaluated using DNA binding assay. Similar procedures were followed for SIRT1 gene silencing using SIRT1 siRNA (Santa Cruz Biotechnology). Effects of tiliroside (6 μM) on nitrite, PGE_2_, TNFα and IL-6 production in LPS/IFNγ-stimulated control siRNA and SIRT1-siRNA-transfected BV2 cells were then determined. Transfection efficiency was determined using western blotting.

### Immunoblotting

For western blotting, 20–40 μg of total protein from each sample was subjected to SDS-PAGE under reducing conditions. Proteins were then transferred onto polyvinylidene fluoride (PVDF) membranes (Millipore). The membranes were blocked for 1 h at room temperature and then incubated overnight at 4 °C with primary antibodies. Primary antibodies used were rabbit anti-COX-2 (Santa Cruz), rabbit anti-iNOS (Santa Cruz), rabbit anti-acetyl-p65 (Cell Signalling), rabbit anti-Total-p65 (Cell Signalling), rabbit anti-MAP2(Cell Signalling), rabbit anti-Nrf2 (Santa Cruz), rabbit anti-HO1 (Santa Cruz), rabbit anti-NQO1 (Santa Cruz), rabbit anti-SIRT1 (Santa Cruz) and rabbit anti-actin (Sigma). Primary antibodies were diluted in Tris-buffered saline (TBS), containing 0.1% Tween 20 (TBS-T) and 1 or 5% BSA. Membranes were incubated with the primary antibody overnight at 4 °C. After extensive washing (three times for 15 min each in TBS-T), proteins were detected by incubation with Alexa Fluor 680 goat anti-rabbit secondary antibody (1:10,000; Life Technologies) at room temperature for 1 h. Detection was done using a LICOR Odyssey Imager. All western blot experiments were carried out at least three times.

### Immunofluorescence

Following treatments, cells were fixed with ice-cold methanol (100%) for 15 min at − 20 °C and later washed three times for 5 min with PBS. Later, cells were blocked using 5% BSA blocking solution (containing 10% horse serum in 1× TBS-T) for 60 min at room temperature followed by washing with PBS. Thereafter, the cells were incubated with 1:100 dilution of rabbit anti-mouse MAP2 (Santa Cruz), Nrf2 (Santa Cruz) and SIRT1 (Santa Cruz) antibody for overnight at 4 °C. Following overnight incubation, cells were washed three times with PBS and incubated for 2 h in dark with Alexa Fluor 488-conjugated donkey anti-rabbit IgG (Life Technologies) secondary antibody (1:500). Later, cells were washed with PBS and counterstained with 4′,6 diamidino-2-phenylindole dihydrochloride (50 nm, DAPI; Invitrogen) for 5 min. After rinsing cells with PBS, the excess buffer was removed and gold antifade reagent (Invitrogen) was added. All staining procedures were performed at room temperature. Representative fluorescence images were obtained using EVOS® FLoid® Cell imaging station (Invitrogen).

### BV2 Microglia Conditioned Medium-Induced HT22 Mouse Hippocampal Neurotoxicity

The effect of conditioned medium obtained from microglia on the viability of HT22 cells was measured using the XTT assay (Invitrogen). BV2 cells were pre-treated with tiliroside (2–6 μM) for 30 min and stimulated with LPS (100 ng/ml)/IFNγ (5 ng/ml) for 24 h. Stimulation was terminated by collecting conditioned medium from the cells and centrifuged and stored at − 80 °C. HT22 hippocampal neurons were seeded at a density of 2 × 10^5^ cells/ml in 96-well cell culture plates and incubated at 37 °C. When cells reach confluence, the culture medium was removed and replaced with 100 μl of conditioned medium and further incubated for 24 h. Stimulation was terminated by adding 25 μl of XTT/PMS solution and incubated for 2 h at 37 °C, followed by gentle shaking for few seconds to distribute the orange colour before absorbance was read at 450 nm with a plate reader (Infinite F50, Tecan).

### Cellular ROS Generation

The effect of conditioned medium on levels of intracellular ROS in HT22 neuronal cells was investigated using 2′,7′-dichlorofluorescin diacetate (DCFDA)-cellular reactive oxygen species detection assay kit (Abcam) that uses a cell-permeable fluorogenic dye DCFDA. HT22 hippocampal neurons were seeded at a density of 2 × 10^5^ cells/ml in 96-well cell culture plates and incubated at 37 °C. After 48 h, cells were washed with PBS and stained with 20 μM DCFDA followed by further incubation at 37 °C for 30 min. After incubation, the cells were washed with PBS and thereafter treated with 200 μl of conditioned medium and incubated for further 24 h at 37 °C. Intracellular accumulation of ROS was measured using Polar Star Optima microplate reader (BMG LABTECH) at an excitation wavelength of 485 nm and an emission wavelength of 535 nm.

### DNA Fragmentation Assay in HT22 Neuronal Cells

The DNA fragmentation of HT22 neurons treated with conditioned medium was measured using an ELISA based non-radioactive cellular DNA fragmentation assay kit (Roche Diagnostics), as earlier described [20].

### Calcium Quantification in HT22 Neuronal Cells

Levels of calcium in the cytoplasm of BV2 cells were measured using calcium detection assay kit (Abcam). HT22 neuronal cells were seeded into 24-well at a density of 2 × 10^5^ cells/ml and allowed to settle. At confluence, the medium was replaced with conditioned medium as explained previously and incubated for 24 h at 37 °C. Later, lysates were collected using RIPA lysis buffer followed by centrifugation at 4 °C, 13,500 rpm for 15 min. Fifty microlitres of the lysates was pipetted into 96-well plate followed by 90 μl of chromogenic reagent along with 60 μl of calcium assay buffer, incubated in the dark for 15 min at room temperature. Output was measured using Tecan F50 microplate reader at a wavelength of 575 nm. Concentrations of calcium in the cytoplasmic lysates were calculated by comparing to the standard curve (0–2.0 μg/ml).

### Transfection of ReNcell VM Human Neural Stem Cells with APPSwe Plasmid

ReNcell VM human neural stem cells were differentiated by replacing the culture medium with ReNcell NSC maintenance medium without the growth factors EGF and FGF-2 for 7 days. Differentiation into neurons was confirmed by immunocytochemical staining for βIII-tubulin. Differentiated cells were transfected with 4 μg of pCAX APP Swe/Ind (Addgene plasmid # 30145) and pCAX APP 695 (Addgene plasmid # 30137) plasmids using and 10 μl of Lipofectamine 2000 (Thermo Fisher Scientific) in Opti-MEM according to the manufacturer’s instructions. Following transfection, cells were treated with tiliroside (2–6 μM) for 48 h. Cell viability was evaluated using XTT assay. Cellular DNA fragmentation and ROS production were also measured.

### Statistical Analysis

Statistical analysis was done by one-way analysis of variance (ANOVA) with post-hoc Student Newman-Keuls test (multiple comparisons). Differences were considered significant at *p* < 0.05. For neuroinflammation experiments, designations include ^&^*p* < 0.05, ^&&^*p* < 0.01, ^&&&^*p* < 0.001 compared with untreated control and *p < 0.05, **p < 0.01, ***p < 0.001 in comparison with LPS/IFNγ control. For siRNA experiments, ^θ^*p* < 0.05, ^θθ^*p* < 0.01, ^θθθ^*p* < 0.001 in comparison within the groups of the untreated control. ^$^*p* < 0.05, ^$$^*p* < 0.01, ^$$$^*p* < 0.001 as compared within the groups stimulated with LPS/IFNγ and ^#^*p* < 0.05, ^##^*p* < 0.01, ^###^*p* < 0.001 as compared within the groups pre-treated with tiliroside (6 μM). At least three independent experiments were performed for analysis. Where necessary, original data were converted into % values of control or LPS/IFNγ and mean ± S.E.M. was calculated.

## Results

### Tiliroside-Activated Nrf2/ARE-Associated Antioxidant Proteins in the Microglia

**Fig. 1:**
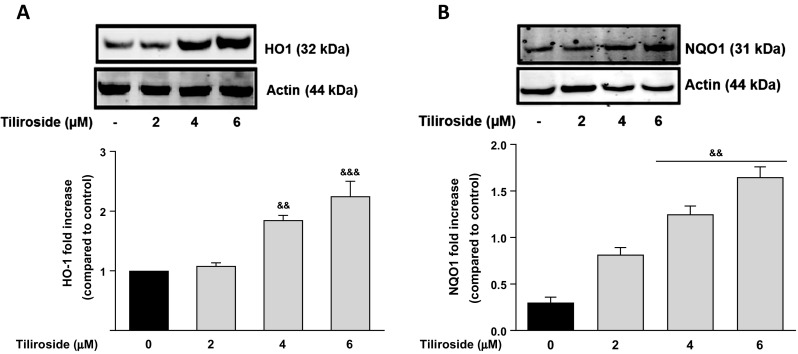
Tiliroside increased levels of HO-1 and NQO1 proteins in BV2 microglia. BV2 cells were treated with various concentrations of tiliroside for 24 h. Cytoplasmic and nuclear lysates were collected and analysed for **a** HO-1 and **b** NQO1 protein expressions using western blot. Tiliroside significantly increased protein levels of HO-1 and NQO1 in the microglia. All values are expressed as mean ± SEM for three independent experiments. Data were analysed using one-way ANOVA for multiple comparisons with post-hoc Student Newman-Keuls test. &*p* < 0.05, &&*p* < 0.01, &&&*p* < 0.001 compared with untreated control

**Fig. 2:**
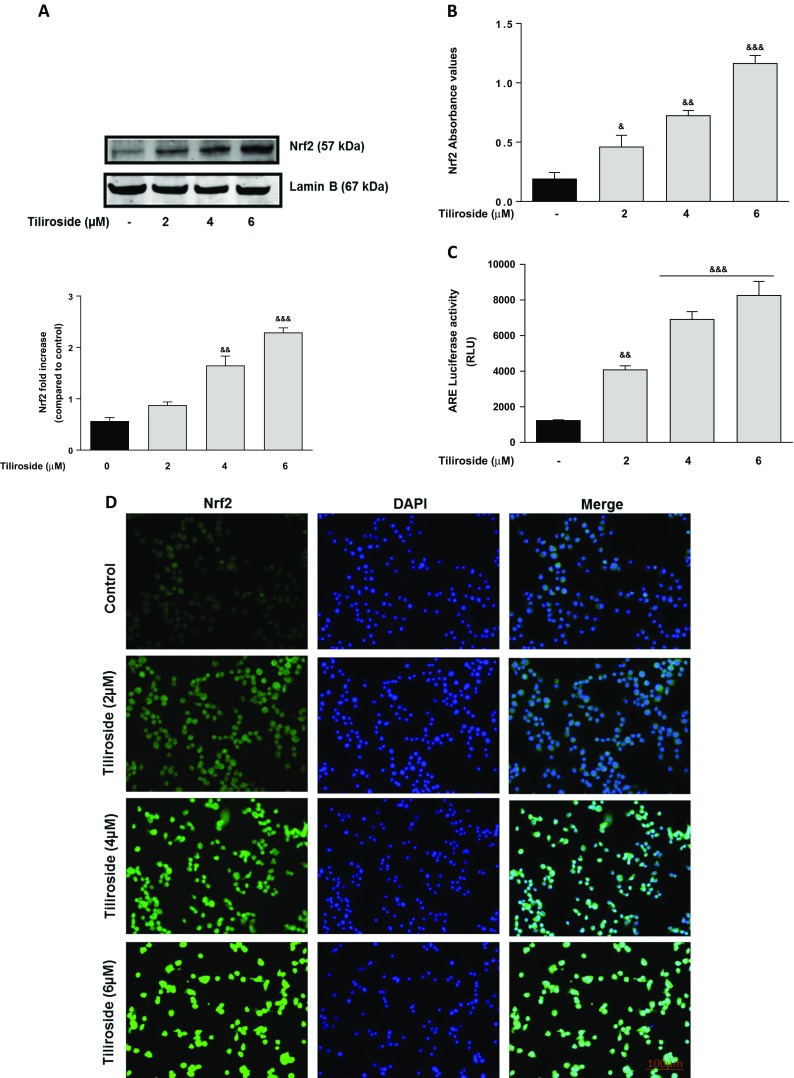
Tiliroside increased Nrf2 protein expression in BV2 microglia. Microglia were treated with tiliroside for 24 h. After that, nuclear lysates were collected and analysed for the activation of Nrf2 using western blot and DNA binding assay. **a** Tiliroside upregulated the protein expression of Nrf2 when incubated for 24 h in microglia. **b** Tiliroside produced a dose-related increase in DNA binding of Nrf2 to immobilised ARE consensus binding site in microglia. **c** Tiliroside activated ARE-luciferase activities transfected with ARE construct in BV2 cells using luciferase reporter gene assay. Microglia cells were transfected with ARE-reporter construct for 18 h. Thereafter, cells were treated with tiliroside for 8 h and luciferase activity was further measured. **d** Immunofluorescence experiments were carried out to detect Nrf2 activation by tiliroside. Nrf2 protein was not detected in untreated cells; however, increasing concentrations of tiliroside activated Nrf2 protein. Cells were counterstained with DAPI and fluorescence images acquired with an EVOS® FLoid® cell imaging station (scale bar = 100 μm) and processed using image J. All values are expressed as mean ± SEM for at least three independent experiments. Data were analysed using one-way ANOVA for multiple comparisons with post-hoc Student Newman-Keuls test. ^&^*p* < 0.05, ^&&^*p* < 0.01, ^&&&^*p* < 0.001 compared with untreated control

The ability of tiliroside to activate antioxidant proteins was investigated in BV2 microglia. Western blot experiments revealed that tiliroside (4 and 6 μM) produced a significant (*p* < 0.01) increase in the expression of HO-1 and NQO1 proteins after 24 h, when compared to untreated microglial cells (Fig. 1). Western blot results showed that treatment of BV2 microglia with 2 μM tiliroside did not result in a significant increase in Nrf2 protein. However, on increasing the concentrations of tiliroside to 4 and 6 μM, there were significant and dose-dependent increases in Nrf2 protein in the nucleus (Fig. 2a). These results were confirmed with immunofluorescence experiments which showed that there were relatively low levels of Nrf2 in untreated cells. In the presence of tiliroside (2–6 μM), there was an increase in Nrf2 immunofluorescence, suggesting that the compound can increase nuclear accumulation of Nrf2 in BV2 microglia.

Further experiments using Nrf2-DNA binding assay revealed that the tiliroside produced a dose-related increase in DNA binding of Nrf2 compared to untreated cells (Fig. 2b). Also, a luciferase reporter gene assay was performed to verify whether the effects of tiliroside were mediated through activation of the antioxidant responsive elements, which is under the control of a promoter containing the ARE consensus. As shown in Fig. 2c, tiliroside (2–6 μM) produced a significant and dose-dependent increase in the ARE-luciferase activity in BV2 microglia.

### Inhibition of Neuroinflammation by Tiliroside Is Dependent on Nrf2

**Fig. 3:**
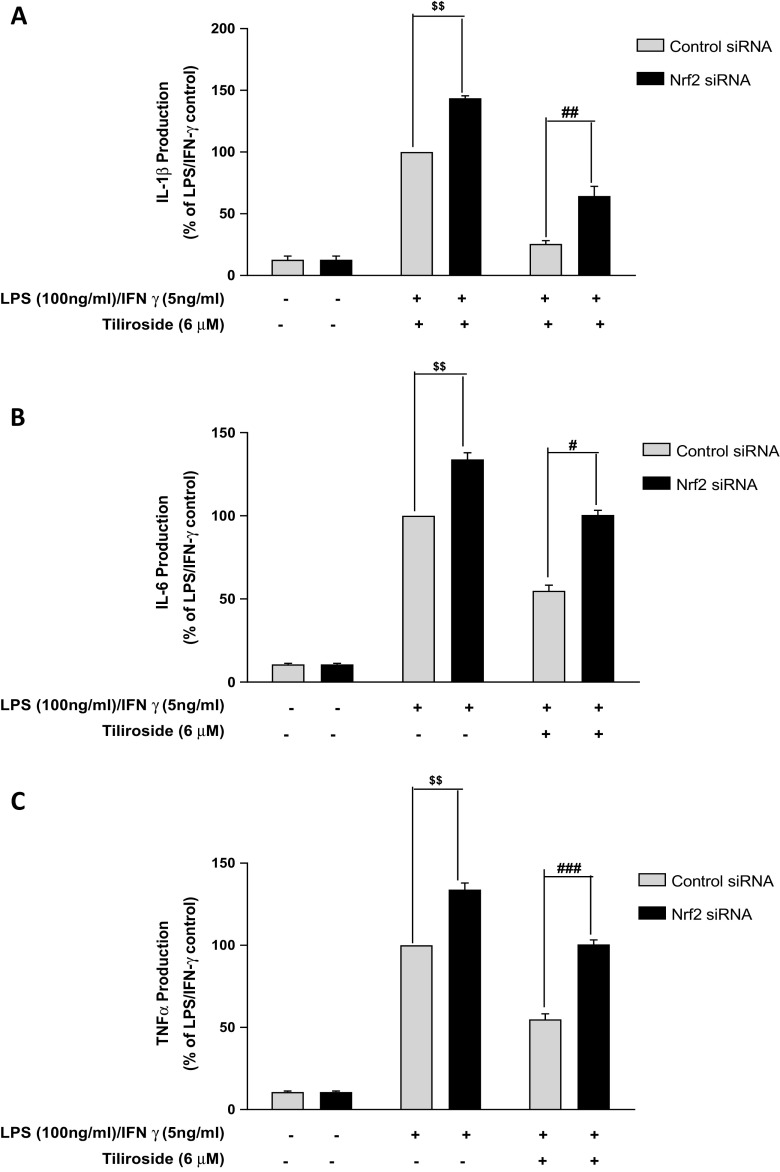
Inhibitory actions of tiliroside on pro-inflammatory cytokine are dependent on Nrf2 activity. Both Nrf2 siRNA and control siRNA-transfected BV2 cells were incubated with tiliroside (6 μM) prior to stimulation with LPS and IFNγ for 24 h. Subsequently, culture supernatants were analysed to detect the levels of IL-1β (**a**), IL-6 (**b**) and TNFα (**c**) using ELISA. Inhibition of IL-1β, IL-6 and TNFα was observed in control siRNA-transfected microglia; however, these inhibitory actions were disappeared in Nrf2 knockout cells. All values are expressed as mean ± SEM for at least three independent experiments. Data were analysed using one-way ANOVA for multiple comparisons with post-hoc Student Newman-Keuls test. ^θ^*p* < 0.05, ^θθ^*p* < 0.01, ^θθθ^*p* < 0.001 as compared within the groups of the untreated control. $*p* < 0.05, $$*p* < 0.01, $$$*p* < 0.001 as compared within the groups stimulated with LPS/IFNγ and #*p* < 0.05, ##*p* < 0.01, ###*p* < 0.001 as compared within the groups pre-treated with tiliroside (6 μM)

In our previous study, we have shown that tiliroside inhibited neuroinflammation in the activated microglia. It is possible that Nrf2 antioxidant protective mechanisms may partly mediate this activity. Therefore, we used RNAi to determine whether the anti-neuroinflammatory effect of tiliroside was dependent on Nrf2 activation in the microglia. As shown in Fig. 3a, tiliroside (6 μM) produced a significant reduction in LPS/IFNγ-induced IL-1β production in control siRNA-transfected cells. In contrast, the IL-1β inhibitory effects of the compound were significantly reversed in Nrf2 siRNA-transfected cells. Further experiments showed that tiliroside inhibited LPS/IFNγ-induced IL-6 and TNFα production in control siRNA cells. However, these inhibitory effects were significantly abolished in the absence of Nrf2 gene (Fig. 3b, c).

**Fig. 4:**
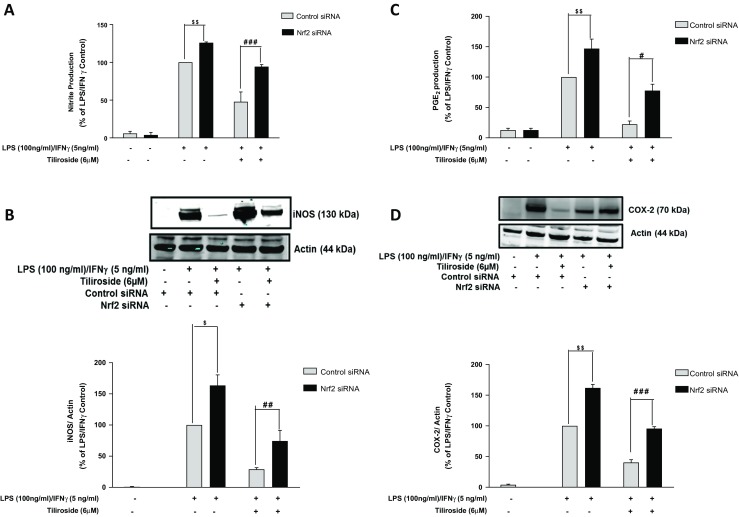
Nrf2 knockout in BV2 microglia reversed suppressive effects of tiliroside on LPS/IFNγ-induced iNOS/nitrite production and COX-2/PGE_2_ production. BV2 microglia were transfected with Nrf2 siRNA and control siRNA. After that, cells were treated with tiliroside (6 μM) prior to LPS and IFNγ stimulation for 24 h. Subsequently, supernatants and cytoplasmic lysates were analysed for nitrite production and iNOS protein expression. **a** Inhibition of nitrite production, **b** iNOS protein expression, **c** PGE_2_ production and **d** COX-2 protein expression was observed in control siRNA-transfected microglia; however, these inhibitory actions were disappeared in Nrf2 knockout cells. All values are expressed as mean ± SEM for at least three independent experiments. Data were analysed using one-way ANOVA for multiple comparisons with post-hoc Student Newman-Keuls test. ^θ^*p* < 0.05, ^θθ^*p* < 0.01, ^θθθ^*p* < 0.001 as compared within the groups of the untreated control. $*p* < 0.05, $$*p* < 0.01, $$$*p* < 0.001 as compared within the groups stimulated with LPS/IFNγ and #*p* < 0.05, ##*p* < 0.01, ###*p* < 0.001 as compared within the groups pre-treated with tiliroside (6 μM)

Encouraged by these results, we also investigated the effect of Nrf2 knockout on the suppression of nitrite/iNOS and PGE_2_/COX-2 by tiliroside in the activated BV2 microglia. Results show that tiliroside significantly inhibited nitrite and prostaglandin E2 (PGE_2_) production in LPS/IFNγ-activated control siRNA-transfected cells. Interestingly, in Nrf2-silenced BV2 cells, these inhibitory effects of the compound were significantly (*p* < 0.05) reversed (Fig. 4a–c). Western blot experiments show that tiliroside (6 μM) significantly attenuated the increase in iNOS and COX-2 protein expression in control siRNA BV2 cells activated with LPS/IFNγ. However, when Nrf2 gene was silenced, these inhibitory effects were significantly reversed (Fig. 4d).

**Fig. 5:**
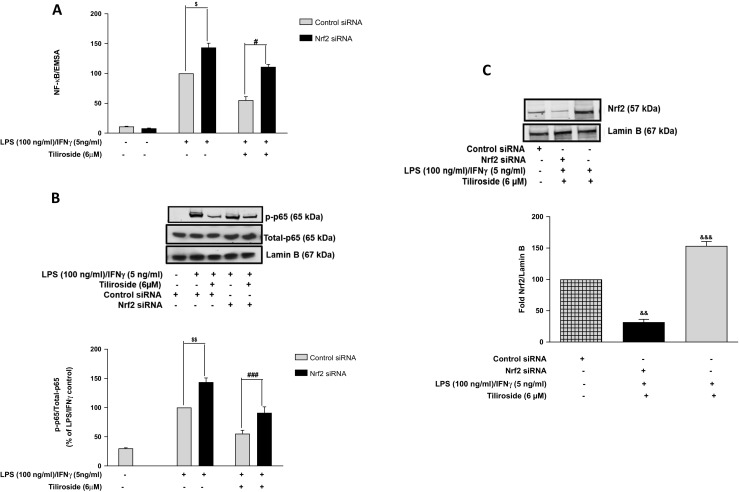
Inhibitory actions of tiliroside on NF-κB were dependent on Nrf2 activity in activated BV2 microglia. Nrf2-silenced BV2 cells were pre-treated with tiliroside prior to stimulation with LPS/IFNγ for 24 h. Subsequently, nuclear and cytoplasmic lysates were collected and subjected to ELISA-based EMSA and western blot. Tiliroside inhibited **a** NF-κB DNA binding and **b** phosphorylation of p65 in BV2 microglia that were transfected with control siRNA. However, these inhibitory effects of the compound disappeared in the absence of Nrf2. **c** Assessment of transfection and Nrf2 knockdown efficiency. Nuclear lysates were collected and assessed for Nrf2 protein expression using western blot. Nrf2 protein was significantly knocked down compared to control siRNA in the microglia. All values are expressed as mean ± SEM for at least three independent experiments. Data were analysed using one-way ANOVA for multiple comparisons with post-hoc Student Newman-Keuls test. ^θ^*p* < 0.05, ^θθ^*p* < 0.01, ^θθθ^*p* < 0.001 as compared within the groups of the untreated control. $*p* < 0.05, $$*p* < 0.01, $$$*p* < 0.001 as compared within the groups stimulated with LPS/IFNγ and #*p* < 0.05, ##*p* < 0.01, ###*p* < 0.001 as compared within the groups pre-treated with tiliroside (6 μM)

The impact of Nrf2 gene silencing on NF-κB inhibitory activity of tiliroside in BV2 microglia was also investigated. As shown in Fig. 5a, tiliroside significantly inhibited DNA binding of NF-κB in control siRNA-transfected cells. Interestingly, this DNA binding inhibitory activity of the compound was reversed in Nrf2 siRNA-transfected cells. Further experiments showed that the combination of LPS and IFNγ upregulated the phosphorylation of p65 in control siRNA microglia. Whereas, in Nrf2 siRNA-transfected cells, inhibitory actions of the compound were completely abolished (Fig. 5b) suggesting that Nrf2 activation possibly contributes to the anti-neuroinflammatory actions of tiliroside. Western blotting experiments further revealed that there was a marked deletion of nuclear Nrf2 protein in Nrf2 siRNA-transfected BV2 cells (Fig. 5c), suggesting that the Nrf2 gene was significantly knocked out in microglia.

### Tiliroside Inhibits Acetylation of NF-κB-p65 in LPS/IFNγ-Activated Microglia

**Fig. 6:**
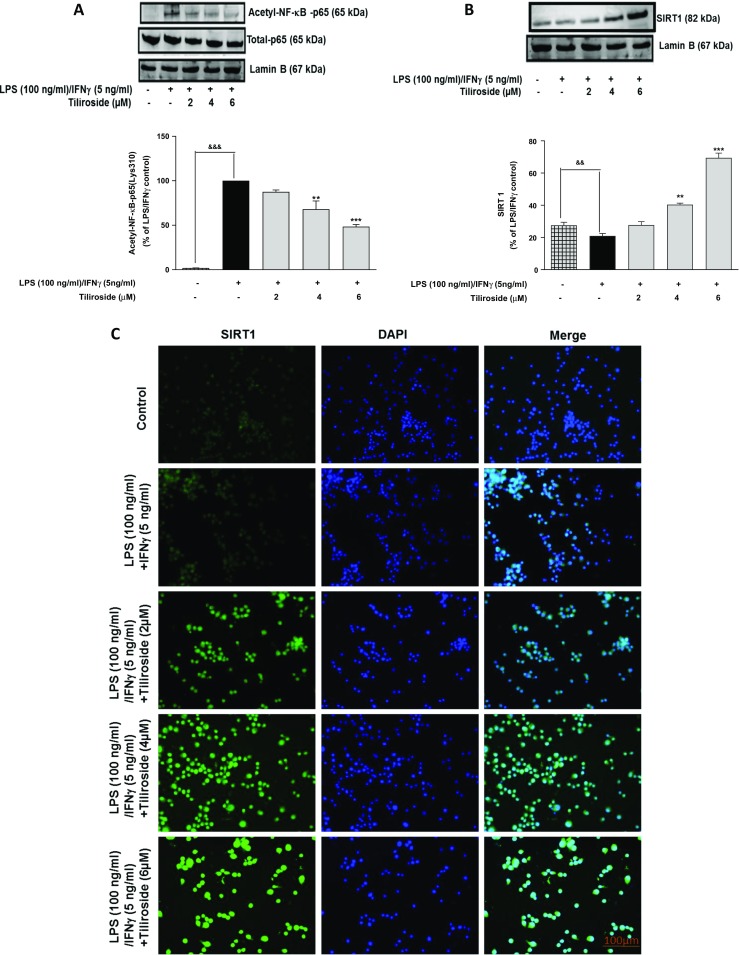
Tiliroside inhibited acetylation of NF-κB-p65 and activated SIRT1 in LPS/IFNγ-treated BV2 microglia. BV2 Cells were treated with LPS/IFNγ in the presence or absence of the tiliroside for 60 min. Later, nuclear lysates were collected and western blotting was done for Acetyl-NF-κB-p65 and Total-NF-κB-p65 protein detection. **a** Tiliroside inhibited acetylation of NF-κB-p65 in LPS/IFNγ-activated microglia compared to Total-p65. **b** BV2 cells were pre-incubated with tiliroside (2–6 μM) for 30 min, following stimulation with LPS/IFNγ for 24 h. Nuclear lysates were collected and subjected to western blotting to detect SIRT1 expression. Results show that the SIRT1 expression was downregulated in neuroinflammation, where tiliroside significantly reversed this inhibition. Lamin B was used as a loading control. **c** BV2 cells were pre-incubated with tiliroside (2–6 μM) for 30 min, following stimulation with LPS/IFNγ for 24 h. Later, cells were fixed, blocked and stained with SIRT1 antibody. Cells were counterstained with DAPI and fluorescence images acquired with an EVOS® FLoid® cell imaging station (scale bar = 100 μm) and processed using image J. Results show that the SIRT1 expression was downregulated in LPS/IFNγ-treated cells, where tiliroside significantly reversed this inhibition. All values are expressed as mean ± SEM for three independent experiments. Data were analysed using one-way ANOVA for multiple comparisons with post-hoc Student Newman-Keuls test. ^&^*p* < 0.05, ^&&^*p* < 0.01, ^&&&^*p* < 0.001 compared with untreated control and *p < 0.05, **p < 0.01, ***p < 0.001 compared to LPS/IFNγ

Compounds that inhibit the activation of NF-κB signalling pathway in hyperactive microglia have been shown to block the expression of acetylated-NF-κB-p65 [21, 22]. Previously, we showed that tiliroside blocked neuroinflammation via NF-κB signalling. Therefore, its effect on LPS/IFNγ-induced acetyl-NF-κB-p65 was investigated in this study. The combination of LPS and IFNγ markedly increased the expression of acetyl-NF-κB-p65 in microglia when compared to unstimulated cells. Interestingly, this upregulation of acetyl-NF-κB-p65 was significantly attenuated by tiliroside treatment, while at 2 μM compound did not show significant effect (Fig. 6a).

### Tiliroside Activates SIRT1 in BV2 Microglia

SIRT1 is a class III histone deacetylase enzyme that has been shown to inhibit NF-κB signalling by deacetylation of NF-κB-p65 subunit at Lysine 310 [23, 24]. Encouraged by earlier observations showing that tiliroside blocked the accumulation of LPS/IFNγ-induced acetyl-NF-κB-p65 in microglia, the effect of tiliroside was next investigated against SIRT1 expression in LPS/IFNγ-activated microglia. Stimulation of BV2 microglia with a combination of LPS and IFNγ resulted in a decrease in the expression of SIRT1 (Fig. 6b). With 2 μM of tiliroside, there was no significant effect on SIRT1 expression. However, pre-treatment with tiliroside (4 and 6 μM) resulted in a significant activation of SIRT1 protein compared with LPS/IFNγ control. These results were further confirmed with immunofluorescence experiments which showed that there were relatively low levels of SIRT1 in both untreated and LPS/IFNγ-treated cells. Interestingly, in the presence of tiliroside (2–6 μM), there was an increase in SIRT1 immunofluorescence, suggesting that the compound can increase nuclear accumulation of SIRT1 in BV2 microglia.

**Fig. 7:**
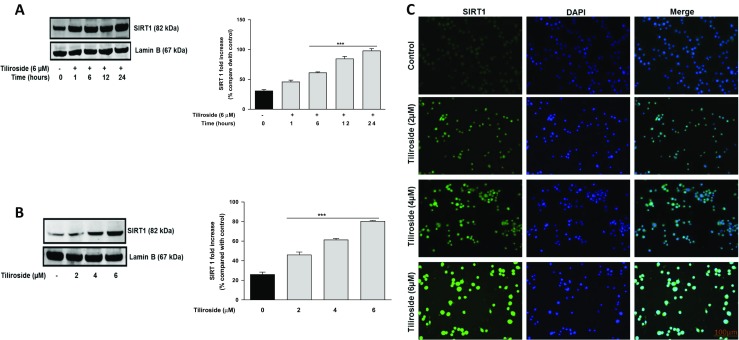
Tiliroside upregulated SIRT1 expression in BV2 microglia. Time point experiments was done by treating BV2 microglia with tiliroside 6 μM and incubated for different time points. Later, nuclear lysates were collected and subjected to western blotting. **a** Tiliroside upregulated SIRT1 expression in microglia. **b** Also, western blot results showed that tiliroside dose-dependently increased the expression of SIRT1 at 24 h in microglia. Lamin B was used as a loading control. **c** BV2 cells were treated with increasing concentrations of tiliroside for 24 h. Later, cells were fixed, blocked and stained with SIRT1 antibody. Cells were counterstained with DAPI and fluorescence images acquired with an EVOS® FLoid® cell imaging station (scale bar = 100 μm) and processed using image J. All values are expressed as mean ± SEM for three independent experiments. Data were analysed using one-way ANOVA for multiple comparisons with post-hoc Student Newman-Keuls test. ^&^*p* < 0.05, ^&&^*p* < 0.01, ^&&&^*p* < 0.001 compared with untreated control and *p < 0.05, **p < 0.01, ***p < 0.001 compared to LPS/IFNγ

Later, we investigated direct effects of the compound on SIRT1 activation in microglia. Figure 7a shows time-dependent activation of SIRT1 in the presence of tiliroside; however, the highest expression was observed at 24 h, compared to untreated cells. Western blotting results show that SIRT1 was significantly (*p* < 0.001) and dose-dependently activated by tiliroside in the microglia (Fig. 7b). Immunofluorescence experiments further revealed that there was no fluorescence in untreated cells, which shows that there was no expression of SIRT1. However, when BV2 cells were incubated with tiliroside (2–6 μM), there was a dose-dependent increase in the expression of SIRT1, suggests that tiliroside activated SIRT1 in microglia.

### Anti-neuroinflammatory Effects of Tiliroside Are Independent of SIRT1 Activity

**Fig. 8:**
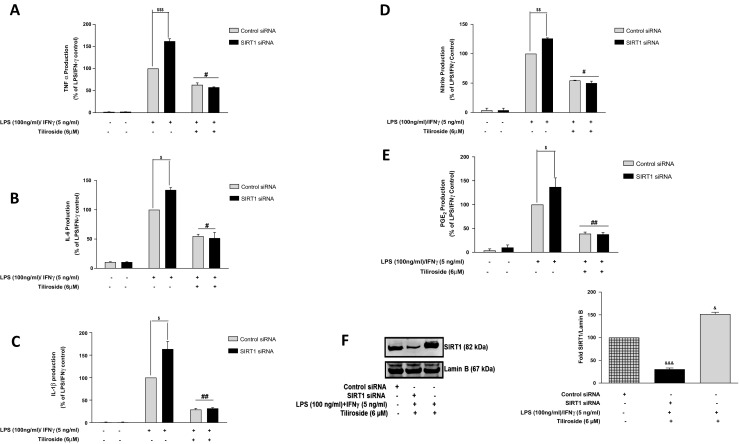
SIRT1 gene knockdown in BV2 microglia did not affect neuroinflammation inhibitory effects of tiliroside. SIRT1 siRNA and control siRNA-transfected BV2 cells were pre-treated with tiliroside for 30 min prior to stimulation with LPS/IFNγ for 24 h. Subsequently, culture supernatants were analysed for **a** TNFα, **b** IL-6, **c** IL-1β, **d** nitrites and **e** PGE_2_ production using ELISA, Griess assay and PGE_2_ by EIA assay kits. Results revealed that tiliroside significantly inhibited the production of pro-inflammatory cytokines in control siRNA cells. However, the inhibitory effects of the compound are not reversed in SIRT1 siRNA BV2 cells. **f** Control siRNA and SIRT1 siRNA-transfected BV2 microglia were treated with tiliroside (6 μM) for 30 min prior to stimulation with LPS/IFNγ for 24 h. Nuclear lysates were collected and assessed for SIRT1 expression using western blot. SIRT1 protein was successfully knocked out compared to control siRNA in the microglia. All values are expressed as mean ± SEM for at least three independent experiments. Data were analysed using one-way ANOVA for multiple comparisons with post-hoc Student Newman-Keuls test. ^θ^*p* < 0.05, ^θθ^*p* < 0.01, ^θθθ^*p* < 0.001 as compared within the groups of the untreated control. $*p* < 0.05, $$*p* < 0.01, $$$*p* < 0.001 as compared within the groups stimulated with LPS/IFNγ and #*p* < 0.05, ##*p* < 0.01, ###*p* < 0.001 as compared within the groups pre-treated with tiliroside (6 μM)

To determine whether nuclear SIRT1 contributed to the suppressive effects of tiliroside on LPS/IFNγ-activated inflammatory responses, BV2 cells were transiently transfected with control siRNA and SIRT1 siRNA. Results show that tiliroside significantly inhibited the production of cytokines in control siRNA cells; interestingly, in SIRT1 silenced cells, the inhibitory activity of the compound was not reversed suggesting that the activity of the compound is independent of SIRT1 activation in microglia (Fig. 8a–c). Further experiments were done to investigate the effects of the compound against the production of NO and PGE_2_ in SIRT1-silenced LPS/IFNγ-treated BV2 microglia. Microglial SIRT1-deficient microglia produced elevated nitrites and PGE_2_ production; however, no reversal of this upregulation was observed in the presence of tiliroside. These results seem to be consistent with data on cytokines suggesting that anti-neuroinflammatory activities of the compound are not SIRT1 mediated (Fig. 8d, e). The efficiency of SIRT1 gene knockdown in BV2 cells was further assessed using western blotting experiments. Results show that cells that are transfected with control siRNA significantly expressed SIRT1. However, following transfection of microglia with SIRT1 siRNA, there was a marked deletion of nuclear SIRT1 protein in the cells (Fig. 8f).

### Tiliroside Inhibited Neuroinflammation-Mediated Neurotoxicity in HT22 Neurons

**Fig. 9:**
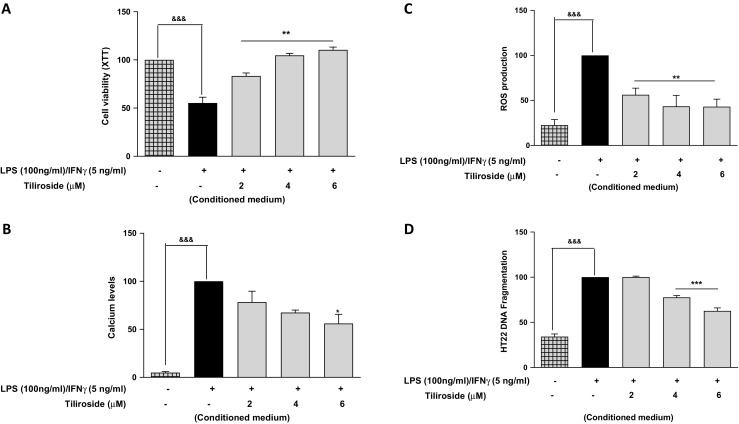
Tiliroside inhibited neuroinflammation-mediated neurotoxicity in HT22 cells. HT22 neurons were incubated with conditioned culture medium obtained from BV2 microglia that were stimulated with LPS and IFNγ. Subsequently, other wells containing HTT22 neurons were exposed to conditioned medium obtained from BV2 cells that were pre-treated with increasing concentrations of tiliroside (2–6 μM) prior to stimulation with LPS/IFNγ for 24 h. **a** XTT assay was carried out to assess the viability of HT22 neurons. Results showed that tiliroside reversed the neuroinflammation-induced HT22 neuronal death. **b** Calcium accumulation assay was carried out in HT22 neurons. Results show that microglia-induced neuroinflammation increased calcium levels in HT22 neurons. Culture medium from BV2 cells pre-treated with tiliroside reduced calcium accumulation in HT22 neurons. **c** ROS production in HT22 neurons was determined by using DCFDA-assay kit. Results show that conditioned medium from microglia that were pre-treated with tiliroside dose-dependently reduced ROS generation in HT22 neurons. **d** DNA fragmentation assay was performed using ELISA based DNA fragmentation assay kit. Results showed that tiliroside (2–6 μM) reduced neuroinflammation-induced DNA fragmentation of HT22 neurons. All values are expressed as mean ± SEM for three independent experiments. Data were analysed using one-way ANOVA for multiple comparisons with post-hoc Student Newman-Keuls test. ^&^*p* < 0.05, ^&&^*p* < 0.01, ^&&&^*p* < 0.001 in comparison with untreated control and *p < 0.05, **p < 0.01, ***p < 0.001 compared to LPS/IFNγ

It is now well established that microglia-induced neuroinflammation is involved in neuronal damage in the brain causing neurodegenerative diseases like AD and PD [25, 26]. The neurotoxic effects of conditioned medium collected from microglia that were stimulated with LPS and IFNγ were tested on HT22 neuronal cells. Results from XTT assay showed that there was a marked reduction in the viability of HT22 neurons when exposed to conditioned medium from the microglia that were incubated with LPS and IFNγ for 24 h (Fig. 9a). However, culture medium collected from BV2 cells that were pre-treated for 30 min with increasing concentrations of tiliroside (2–6 μM) before the stimulation with LPS/IFNγ significantly (*p* < 0.01) inhibited neuronal toxicity induced by neuroinflammation (Fig. 9a).

We next investigated the effects of conditioned medium on neuronal calcium accumulation. Results show that the conditioned culture medium from BV2 cells that were stimulated with LPS/IFNγ induced high levels of calcium (*p* < 0.001) in HT22 neurons. However, incubation of HT22 cells with conditioned medium collected from BV2 cells that are pre-treated with tiliroside showed a minimal effect at concentrations of 2 and 4 μM. Surprisingly culture medium from microglia that were pre-treated with 6 μM tiliroside significantly reduced (*p* < 0.01) calcium accumulation in HT22 neurons (Fig. 9b). Incubation of HT22 cells with conditioned culture medium collected from LPS and IFNγ stimulated BV2 cells significantly increased (*p* < 0.001) intracellular ROS production compared to the cells that were incubated with culture medium collected from control BV2 microglia. Generation of ROS was significantly inhibited (*p* < 0.01) when neurons were incubated with conditioned medium from microglia cells that were pre-treated with compound in a concentration-dependent manner (Fig. 9c). Later, we investigated the effects of conditioned medium on neuronal DNA fragmentation. Results in Fig. 9d show that neuroinflammation increased DNA fragmentation of HT22 neurons compared to the cells treated with culture medium collected from control BV2 microglia. Interestingly, conditioned medium from microglial cells that were pre-treated with tiliroside 2 μM has no significant inhibitory effect on DNA fragmentation, however at 4 and 6 μM showed a dose-dependent reduction in the DNA fragmentation of HT22 neuronal cells.

**Fig. 10:**
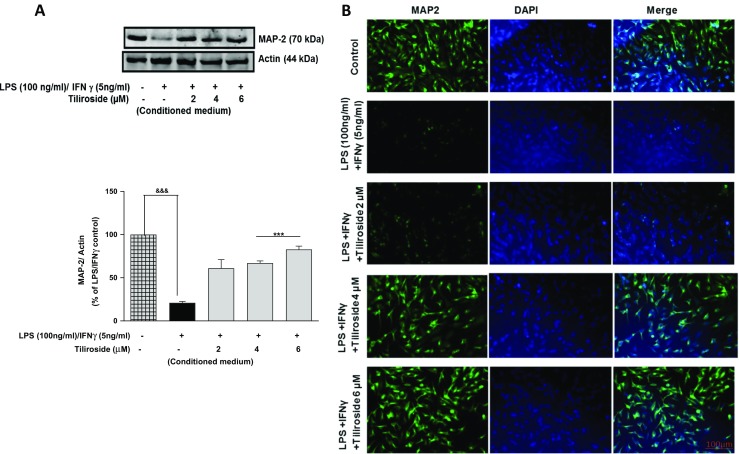
Tiliroside reversed neuroinflammation-induced MAP2 expression in HT22 cells. HT22 neurons were incubated with conditioned culture medium obtained from BV2 microglia that were stimulated with LPS and IFNγ. Subsequently, neurons in other wells were incubated with conditioned medium obtained from BV2 cells that were pre-treated with increasing concentrations of tiliroside (2–6 μM) and stimulated with LPS/IFNγ for 24 h. **a** Cytoplasmic lysates were collected and subjected to western blotting to assess the expression of MAP2 protein. Results showed that tiliroside dose-dependently increased MAP2 protein expression in HT22 neurons. **b** Immunofluorescence experiments were done to investigate the effects of microglia-induced neuroinflammation on the expression of MAP2 protein. HTT22 neuronal cells were treated with microglial conditioned medium for 24 h. Later, cells were fixed, blocked and stained with MAP2 antibody for overnight. The following day, cells were washed and incubated with secondary antibody for 2 h in the dark. Cells were counterstained with DAPI and fluorescence images acquired with an EVOS® FLoid® cell imaging station (scale bar = 100 μm) and processed using image J. Results revealed that tiliroside increased the expression of MAP2 protein in HT22 neurons. All values are expressed as mean ± SEM for three independent experiments. Data were analysed using one-way ANOVA for multiple comparisons with post-hoc Student Newman-Keuls test. ^&^*p* < 0.05, ^&&^*p* < 0.01, ^&&&^*p* < 0.001 compared with untreated control and *p < 0.05, **p < 0.01, ***p < 0.001 compared to LPS/IFNγ

The conditioned medium obtained from BV2 microglia that were stimulated with LPS/ IFNγ was tested against MAP2 protein expression in HT22 neurons. Western blotting results show reduced MAP2 expression in neurons when incubated with culture medium from activated microglia. Surprisingly, at 2 μM the effect of the compound was minimal; however, conditioned medium from the microglia that were pre-treated with tiliroside significantly increased (*p* < 0.001) MAP2 protein expression (Fig. 10a). Immunofluorescence experiment was done to further confirm the effect of microglia-induced neuroinflammation on the expression of MAP2 protein in HT22 neurons. Figure 10b shows that neuroinflammation reduced MAP2 protein expression. However, conditioned culture medium from BV2 cells that were pre-incubated with tiliroside (2–6 μM) increased the expression of MAP2 protein in HT22 neurons.

### Treatment with Tiliroside Prevented APPSwe-Induced Toxicity in Differentiated ReNcell VM Human Neural Stem Cells

**Fig. 11:**
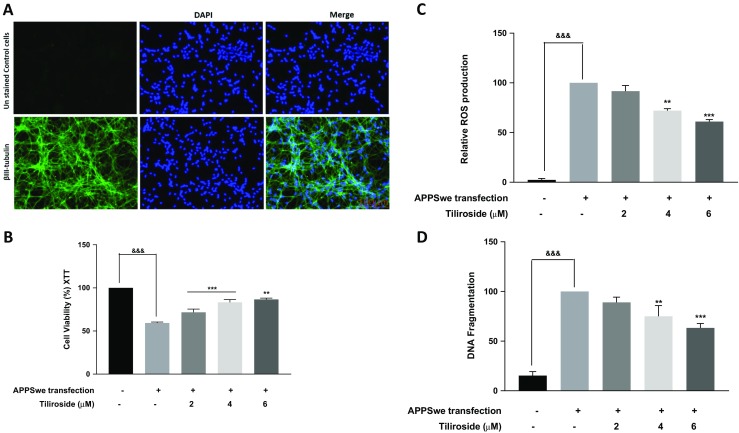
Tiliroside prevented APPSwe-induced neurotoxicity in ReNcell VM human neural stem cells. The APPSwe plasmid was transfected into the ReNcell VM human neural cells and XTT, DNA fragmentation and ROS assay were carried out to assess the neuroprotective effects of the compound. **a** ReNcell differentiation was confirmed by βIII-tubulin immunostaining. **b** Results showed that there was a marked reduction in the viability of neural stem cells when transfected with APPSwe after 48 h. However, pre-treatment with tiliroside for significantly inhibited neuronal death. **d** Neurons that were transfected with APPSwe increased DNA fragmentation compared to the control cells after 24 h. Interestingly, tiliroside exhibited a dose-dependent reduction in the DNA fragmentation of human neuronal cells. **c** Transfection of ReNcell neurons with APPSwe significantly increased intracellular ROS production compared to the untreated cells and tiliroside blocked generation of ROS in a concentration-dependent manner after 48 h. All values are expressed as mean ± SEM for three independent experiments. Data were analysed using one-way ANOVA for multiple comparisons with post-hoc Student Newman-Keuls test. ^&^*p* < 0.05, ^&&^*p* < 0.01, ^&&&^*p* < 0.001 compared with untreated control and *p < 0.05, **p < 0.01, ***p < 0.001 compared to APPSwe transfected cells

The APPSwe plasmid was transfected into differentiated ReNcell VM human neural cells to establish an in vitro model of Alzheimer’s disease. The differentiation of ReNcell stem cells into neurons was confirmed by βIII-tubulin immunostaining (Fig. 11a). Results from XTT assay showed that there was a marked reduction in the viability of ReNcell VM human neural stem cells when transfected with APPSwe (Fig. 11b). However, pre-treatment with tiliroside for 48 h significantly (*p* < 0.01) inhibited neuronal death. Several studies in the past have highlighted that the APPSwe transfection will increase neuronal accumulation of Amyloid-β (Aβ) and ROS [27–29]. In the present study, transfection of ReNcell neurons with APPSwe significantly increased (*p* < 0.001) intracellular ROS production compared to the untreated cells. Generation of ROS was significantly inhibited (*p* < 0.001) when neurons were incubated with compound in a concentration-dependent manner (Fig. 11c). We further investigated the effects of APPSwe-induced toxicity on ReNcell neuronal DNA fragmentation. Results in Fig. 11d show that neurons that were transfected with APPSwe increased DNA fragmentation compared to the control cells. Interestingly, tiliroside at 2 μM has no significant inhibitory effect on DNA fragmentation, however at 4 and 6 μM compound exhibited a dose-dependent reduction in the DNA fragmentation of human neuronal cells. These results demonstrate that tiliroside protects APPSwe transfected ReNcell VM human neural stem cells suggesting that the compound possesses strong neuroprotective effects against Aβ-induced DNA fragmentation and oxidative stress in neuronal cells.

### Tiliroside Increased Nrf2 and SIRT1 Proteins in HT22 Neuronal Cells

**Fig. 12:**
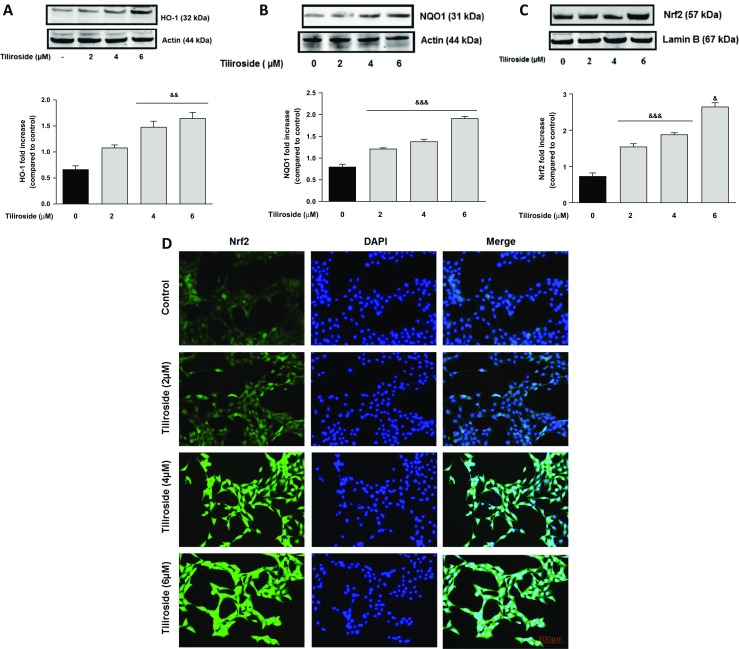
Tiliroside activated HO-1/NQO1 and Nrf2 antioxidant axis in HT22 neurons. HT22 neuronal cells were treated with tiliroside (2–6 μM) for 24 h. Cytoplasmic and nuclear extracts were collected and analysed for **a** HO-1, **b** NQO1 and **c** Nrf2 protein expression using western blot. Tiliroside increased the protein expressions of HO-1, NQO1 and Nrf2 in HT22 neurons. **d** Immunofluorescence experiments were carried out to detect activation of Nrf2 by tiliroside in HT22 neuronal cells. Very low levels of Nrf2 was observed in control cells, while increasing concentrations of the compound induced Nrf2 activation. All values are expressed as mean ± SEM for three independent experiments. Data were analysed using one-way ANOVA for multiple comparisons with post-hoc Student Newman-Keuls test. ^&^*p* < 0.05, ^&&^*p* < 0.01, ^&&&^*p* < 0.001 compared with untreated control

We next investigated whether tiliroside can activate two important antioxidant mechanisms in HT22 neurons. Western blotting results reveal that there was no expression of HO-1 in untreated HT22 neuronal cells. However, incubation with increasing concentrations of tiliroside (2–6 μM) for 24 h showed a marked increase (*p* < 0.01) of HO-1 protein (Fig. 12a). A similar dose-dependent increase (*p* < 0.001) was observed in the levels of NQO1 protein when treated with the compound for 24 h compared with untreated HT22 neuronal cells. Upregulation of antioxidant proteins HO-1 and NQO1 could be attributed to an activation of transcription factor Nrf2 by the compound, therefore tiliroside effect was measured on Nrf2 expression in HT22 neurons. Treatment of HT22 neuronal cells with tiliroside (2–6 μM) for 24 h increased the expression of Nrf2 in a concentration-dependent manner as determined by western blotting experiment. Tiliroside at 2 and 4 μM showed similar effect, however when increased to 6 μM, a significant upregulation (*p* < 0.05) of Nrf2 was noticed compared to control (Fig. 12b, c). Further experiments show a dose-dependent increase in the Nrf2 translocation to nucleus which appears to be concomitant with western blot results. In untreated neuronal cells, no fluorescence was measured, however at 4 and 6 μM, the high appearance of Nrf2 was observed (Fig. 12d).

Accumulating evidence indicate that high levels of SIRT1 exhibit neuroprotective roles in several neurodegenerative diseases [30]. Therefore, the ability of tiliroside to activate SIRT1 in HT22 neuronal cells was investigated in this study. Results show that the compound significantly activated SIRT1 protein in neuronal cells. Notably at 4 and 6 μM, tiliroside dose-dependently upregulated SIRT1 expression compared to untreated cells and at 2 μM the effect of the compound was shown to be minimal (S1). Immunofluorescence experiments show that the tiliroside dose-dependently activated SIRT1 in HT22 neuronal cells compared to control, suggesting that the compound can activate SIRT1 protein in neuronal cells. However, further experiments revealed that neuroprotective effects of tiliroside in HT22 neurons are independent of SIRT1 (S2).

### Nrf2 Mediates Neuroprotective Effects of Tiliroside in HT22 Neurons

**Fig. 13:**
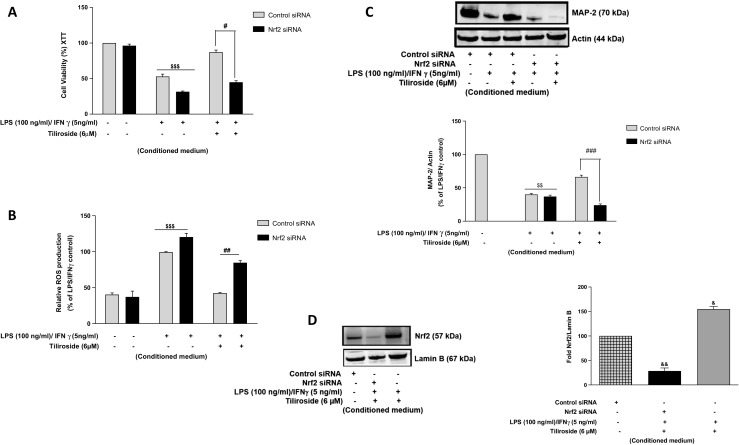
Neuroprotective effects of tiliroside were mediated by Nrf2 in HT22 neurons. Nrf2 gene knockout HT22 neurons were treated with conditioned medium containing LPS (100 ng/ml)/IFNγ (5 ng/ml) and tiliroside (6 μM) for 24 h. Thereafter, **a** XTT and **b** ROS generation assays were carried out. Results show that conditioned medium contained LPS and IFNγ pre-treated with tiliroside possesses neuroprotective effects that are mediated by Nrf2. **c** Subsequently, cytoplasmic extracts were collected from HT22 neurons and subjected to western blotting to assess MAP-2 protein expression. **d** Control siRNA and Nrf2 siRNA-transfected HT22 neuronal cells were treated with tiliroside 6 μM for 24 h. Nuclear extracts were collected and assessed for Nrf2 protein expression using western blot. Nrf2 protein was significantly knocked down compared to control siRNA in HT22 neuronal cells. All values are expressed as mean ± SEM for at least three independent experiments. Data were analysed using one-way ANOVA for multiple comparisons with post-hoc Student Newman-Keuls test. ^θ^*p* < 0.05, ^θθ^*p* < 0.01, ^θθθ^*p* < 0.001 as compared within the groups of the untreated control. $*p* < 0.05, $$*p* < 0.01, $$$*p* < 0.001 as compared within the groups stimulated with LPS/IFNγ and #*p* < 0.05, ##*p* < 0.01, ###*p* < 0.001 as compared within the groups pre-treated with tiliroside (6 μM). *p* < 0.05, ^&&^*p* < 0.01, ^&&&^*p* < 0.001 compared with untreated control

Having shown that tiliroside activates Nr2 signalling pathway in neuronal cells, we further endeavoured to determine whether neuroprotective effects of the drug is dependent on Nrf2 activity. In control siRNA-transfected cells, conditioned medium obtained from activated microglia pre-treated with tiliroside significantly enhanced cell viability, while in Nrf2-silenced cells this effect was abolished (Fig. 13a) suggesting that Nrf2 mediates neuroprotective effects of the compound.

Further experiments on ROS production show that tiliroside significantly inhibited ROS production in control siRNA cells. Interestingly, the ability of the compound to inhibit ROS generation was abolished in Nrf2 siRNA-transfected neuronal cells (Fig. 13b). Based on these results, we next assessed the effect of tiliroside on Nrf2-silenced MAP2 expression in HT22 neurons. Western blot experiments show that tiliroside reversed this suppression of MAP2 protein in control siRNA-transfected neuronal cells (Fig. 13c). In contrast, Nrf2-silenced HT22 neurons exhibited low levels of MAP2 in the presence of tiliroside, which suggests that the compound failed to elevate MAP2 protein levels in the absence of Nrf2. These results show that tiliroside requires Nrf2 to produce neuroprotective effects in HT22 neurons. Efficiency of Nrf2 gene knockdown in HT22 neurons show that HT22 cells that were transfected with control siRNA expressed nuclear Nrf2 protein. However, following transfection of HT22 cells with Nrf2 siRNA, there was a marked deletion of nuclear Nrf2 protein in the cells (Fig. 13d) suggesting that the Nrf2 gene was efficiently knocked out.

## Discussion

The transcription factor Nrf2 protects microglia from cellular stress by activating cytoprotective proteins such as HO-1 and NQO1. Moreover, several in vivo and in vitro disease models revealed that activation of Nrf2 antioxidant mechanism provides protection against neuroinflammation and oxidative stress [31, 32]. Consequently, activation of microglial Nrf2 was thought to be a right approach in resolving neuroinflammatory conditions. Increased consumption of dietary supplements and fruits are associated with the prevention of neuroinflammatory conditions and its mediated neurodegenerative diseases. Tiliroside, a dietary compound, inhibits LPS/IFNγ-mediated neuroinflammation and ROS production in microglia [19]. However, its effect against Nrf2 signalling in BV2 microglia is yet to be investigated.

The role of microglial Nrf2 signalling in neuroinflammation is well documented [33–35]. Antioxidant proteins such as HO-1 and NQO1 are known to play a crucial role in inhibiting neuroinflammation and its mediated neuronal loss [36]. In several in vitro studies, activation of HO-1 and NQO1 was shown to be beneficial against neuroinflammation by inhibiting NADPH oxidase, the primary enzyme responsible for microglial ROS release and from reactive quinones [37–40]. Our results show that tiliroside significantly increased endogenous levels of antioxidant proteins HO-1 and NQO1 in the microglia. These results also suggest that the compound is capable of activating Nrf2 signalling in microglia.

Expression of HO-1 and NQO1 are predominantly regulated by the transcription factor Nrf2 [41]. Following our observation that tiliroside increased protein levels of HO-1 and NQO1, its effect on Nrf2 activation was investigated. Results show that tiliroside increased nuclear accumulation of Nrf2. Further experiments revealed that tiliroside significantly enhanced Nrf2 DNA binding and increased the transcriptional activity of the *cis*-acting ARE, suggesting that tiliroside acts at the transcriptional level of Nrf2 to induce expression of antioxidant proteins HO-1 and NQO1 in the microglia. It therefore appears that tiliroside might be inhibiting neuroinflammation probably by activating Nrf2/HO-1/NQO1 axis in BV2 microglia.

Studies have revealed that Nrf2 activation might attenuate neuroinflammation via modulating NF-κB-mediated inflammatory mediators in microglia [7, 42]. Also, compounds that inhibit NF-κB activation have been shown to activate Nrf2 [43, 44]. For example, the antimalarial drug artemether inhibited LPS-induced neuroinflammation via Nrf2 signalling and those inhibitory effects were lost in the absence of Nrf2 in microglia [20]. Moreover, Nrf2 gene knockout experiments showed that microglia tends to generate excess amounts of inflammatory mediators when stimulated with LPS compared to control, highlighting the protective role of Nrf2 [6, 32, 45]. Results from the current research show that in control siRNA-transfected cells, tiliroside inhibited pro-inflammatory cytokines such as IL-1β, IL-6 and TNFα production in LPS/IFNγ-stimulated microglia. However, these inhibitory activities of tiliroside were significantly abolished following silencing of Nrf2, suggesting that neuroinflammation inhibitory effects of the compound in BV2 microglia are mediated through Nrf2. Similar observations have been made in other studies. For example, kolaviron, a biflavonoid, was shown to inhibit LPS-induced NF-κB signalling in BV2 microglia and also activated Nrf2. Further experiments demonstrated that Nrf2 was required for kolaviron to exhibit neuroinflammation inhibitory effects in microglia [46]. Furthermore, inhibitory effects of tiliroside on NO/iNOS and PGE_2_/COX-2 were reversed in the absence of Nrf2 gene in the microglia, thereby further highlighting that the transcription factor plays a critical role in mediating the anti-inflammatory action of tiliroside.

Studies in various experimental models revealed that several redox-sensitive factors regulate Nrf2 and NF-κB, and lack of Nrf2 was shown to increase nitrosative and oxidative stress leading to increase in cytokine production. To further understand the relationship between Nrf2 and NF-κB in microglia, Nrf2 gene knockout BV2 cells were stimulated with a combination of LPS and IFNγ. Results show that the inhibitory effects of the compound on LPS/IFNγ-induced NF-κB activation and DNA binding were significantly reversed in Nrf2 silenced cells. These results suggest that transcription factor Nrf2 mediates the neuroinflammation inhibitory actions of tiliroside in LPS/IFNγ-stimulated microglia by interfering with processes involved in NF-κB activation. This observation explains previous reports of excessive generation of pro-inflammatory cytokines in the absence of Nrf2 protein [47, 48]. This is the first report linking the neuroinflammation inhibitory property of tiliroside in LPS/IFNγ-activated microglia to Nrf2 activity.

**Fig. 14:**
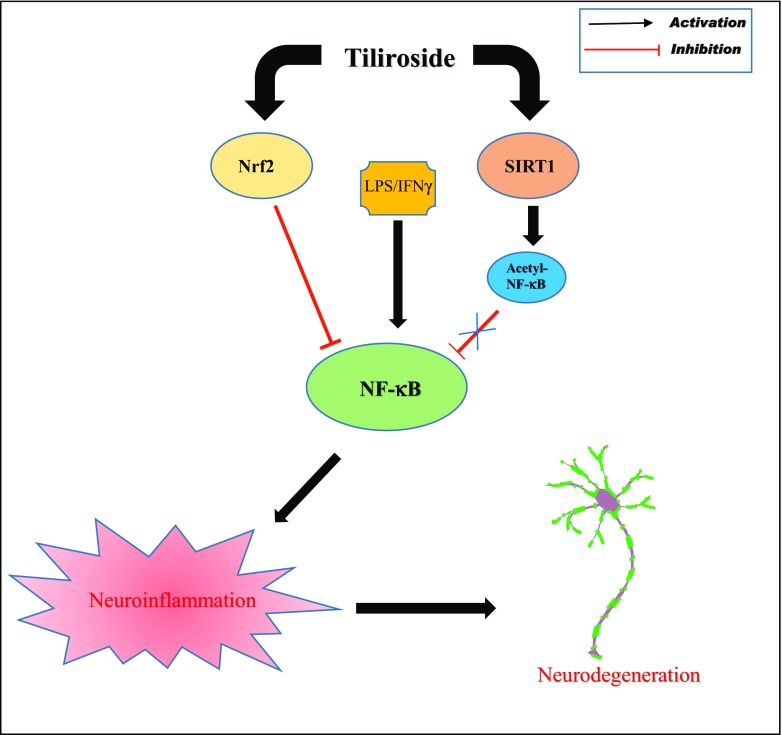
Neuroinflammation inhibition and neuroprotection effects of tiliroside are dependent on Nrf2, but independent of SIRT1

Acetylation of nuclear NF-κB-p65 subunit regulates various functions of NF-κB such as DNA binding affinity and transcriptional activation of pro-inflammatory genes. In the nucleus, SIRT1 deacetylates NF-κB subunit and blocks the binding of acetylated-NF-κB-p65 to the promotor regions of the DNA. Previous studies demonstrated that compounds which activate SIRT1 will reduce the acetylation of NF-κB-p65 and further inhibit the transcription of inflammatory mediators in neuroinflammation [14, 18, 49]. In this study, tiliroside increased the expression of SIRT1 in the microglia. In addition, tiliroside inhibited the acetylation of NF-κB-p65 in LPS/IFNγ-stimulated microglia, suggesting that there is a possibility that this compound inhibits NF-κB signalling via accelerating deacetylation of NF-κB-p65. However, investigations in this study demonstrated that neuroinflammation inhibitory action of tiliroside is not mediated by SIRT1. This observation suggests that SIRT1 activation may not be involved in the inhibition of acetylated-NF-κB-p65 by tiliroside in the microglia. A study conducted by Zhu et al. revealed that resveratrol, a potent SIRT1 activator, inhibits NF-κB-mediated inflammation in TNFα-induced NIH/3T3 fibroblast cell line. However, knockdown of SIRT1 gene reduced anti-inflammatory effects of resveratrol [50]. This observation contrasts with our observations, suggesting the involvement of other signalling pathways in SIRT1-mediated anti-neuroinflammatory activity in the microglia (Fig. 14).

Neuroinflammation-related neurotoxicity contributes to the development of neurodegenerative diseases like AD, PD, HD and ALS [25]. Pro-inflammatory mediators such as TNFα, IL-1β and IL-6 released by activated microglia are shown to induce damage to adjacent neurons [51]. TNFα released by activated microglia promotes neuronal damage by inhibiting cell survival mechanisms such as PI3K/AKT pathway and further activates Bcl-2 family members Bad, Bax, and caspase-9 [52]. Also, treatment with IL-1β induced activation of caspases and other apoptosis signalling cascades leading to the death of neurons via increasing the generation of ROS and DNA fragmentation [53, 54]. These observations further indicate that inflammatory mediators from activated microglia lead to neuronal death. In theory, compounds that are shown to inhibit the production of pro-inflammatory cytokines like TNFα, IL-1β and IL-6 would be expected to confer protection against neurotoxicity. This study demonstrated that inhibition of pro-inflammatory mediator release by tiliroside in the activated microglia increased the viability of HT22 neurons by reducing calcium accumulation, ROS generation and DNA fragmentation. From this observation, it appears that tiliroside indirectly protects neurons by blocking the inflammatory responses in the activated microglia. Similar results were obtained in studies conducted by Lee et al., which revealed that tiliroside isolated from *Agrimonia eupatoria* (Rosaceae) exhibits neuroprotective activity against glutamate-induced toxicity in HT22 neurons. We also tested the effect of tiliroside on amyloid-induced neurotoxicity, by transfecting human neural stem cells with APPSwe plasmid and then treating cells with graded concentrations of the compound. Tiliroside prevented the neuronal death in APPSwe-transfected neural stem cells by reducing DNA fragmentation and ROS generation. Similar observation was made in the studies conducted against neuroprotective roles of curcumin [27]. Overall, these observations suggest that the tiliroside may be exerting direct neuroprotective effects against Aβ in neuronal cells.

To further understand the mechanisms involved in the neuroprotective activity of tiliroside, we investigated its effect against Nrf2/HO-1/NQO1 axis and SIRT1 protein expressions in HT22 hippocampal neurons. Tiliroside significantly increased protein levels of Nrf2, as well as HO-1 and NQO1 in HT22 neurons. Similar effects have been shown by other natural antioxidants and small molecule activators of the Nrf2/HO-1 in neuronal cells [32, 41, 55]. Encouraged by these results, we then explored whether the observed neuroprotective activity of tiliroside was mediated by Nrf2 activity in neuroinflammation-induced HT22 neurons. We showed that activities of tiliroside on levels of MAP2 protein and generation of cellular ROS were significantly abolished in Nrf2-silenced neurons, suggesting that Nrf2 activity contributes to the neuroprotective effects of the compound. Emerging evidence suggests that SIRT1 is involved in the regulation of neuronal survival and death through deacetylation of p53 and NF-κB signalling in neuroinflammation-induced neurodegenerative diseases [30, 56]. Therefore, the effect of tiliroside on SIRT1 expression was further examined in HT22 neuronal cells. We demonstrated that tiliroside dose-dependently increased the expression of SIRT1 in HT22 neurons suggesting that there is a possibility that this compound might be acting on multiple signalling pathways to exhibit neuroprotection. In conclusion, this study has established that tiliroside protected BV2 microglia from LPS/IFNγ-induced neuroinflammation and HT22 neuronal toxicity by targeting Nrf2 antioxidant mechanisms. The compound also produced inhibition of NF-κB acetylation through activation of SIRT1, as well as increasing SIRT1 activity in mouse hippocampal neurons. Results from this study have further established the mechanisms involved in the anti-neuroinflammatory and neuroprotective activities of tiliroside.

## Electronic Supplementary Material

Supplementary Fig. 1 **(S1):** Tiliroside upregulated SIRT1 protein expressions in HT22 neuronal cells. (A) Neurons were incubated with tiliroside (2–6 μM) for 24 h. Later, nuclear extracts were collected and analysed for SIRT1 protein expression using western blot. (B) Immunofluorescence experiments were carried out to detect activation of SIRT1 by tiliroside in HT22 cells. Results reveal that very low levels of SIRT1 were observed in untreated cells while increasing concentrations of the compound induced SIRT1 activation and protein expression in HT22 neurons. All values are expressed as mean ± SEM for three independent experiments. Data were analysed using one-way ANOVA for multiple comparisons with post-hoc Student Newman-Keuls test. ^&^*p* < 0.05, ^&&^*p* < 0.01, ^&&&^*p* < 0.001 compared with untreated control. (PPTX 3982 kb)

Supplementary Fig. 2 **(S2):** Neuroprotective activity of tiliroside is independent of SIRT1 protein activation in HT22 neurons. Cells were transfected with SIRT1 siRNA and control siRNA followed by incubation with conditioned medium containing LPS (100 ng/ml)/IFNγ (5 ng/ml) and tiliroside (6 μM) for 24 h. Thereafter, (A) XTT and (B) ROS generation assays were carried out. Results show that both cells that contained control and SIRT1 siRNA exhibited similar outcome. (C) Subsequently, cytoplasmic extracts were collected and subjected to western blotting to assess MAP2 expression. (D) Control siRNA and SIRT1 siRNA-transfected BV2 microglia, treated with tiliroside 6 μM for 24 h. Nuclear extracts were collected and assessed for SIRT1 expression using western blot. SIRT1 protein was significantly knocked down compared to control siRNA in HT22 neuronal cells. All values are expressed as mean ± SEM for at least three independent experiments. Data were analysed using one-way ANOVA for multiple comparisons with post-hoc Student Newman-Keuls test. ^θ^*p* < 0.05, ^θθ^*p* < 0.01, ^θθθ^*p* < 0.001 as compared within the groups of the untreated control. $*p* < 0.05, $$*p* < 0.01, $$$*p* < 0.001 as compared within the groups stimulated with LPS/IFNγ and #*p* < 0.05, ##*p* < 0.01, ###*p* < 0.001 as compared within the groups pre-treated with tiliroside (6 μM). *p* < 0.05, ^&&^*p* < 0.01, ^&&&^*p* < 0.001 compared with untreated control. (PPTX 286 kb)

## Abbreviations

AD: Alzheimer’s disease
ANOVA: Analysis of variance
BACE-1: Beta-site amyloid precursor protein cleaving enzyme 1
CNS: Central nervous system
COX: Cyclooxygenase
DMSO: Dimethyl sulfoxide
FBS: Foetal bovine serum
IκB: Inhibitor of kappa B
IL: Interleukin
IFNγ: Interferon gamma
iNOS: Inducible nitric oxide synthase
LPS: Lipopolysaccharide
MAPK: Mitogen-activated protein kinase
HO-1: Heme oxygenase-1
NO: Nitric oxide
Nrf2: Nuclear factor erythroid 2-related factor 2
NQO1: NAD(P)H quinone dehydrogenase 1
NF-κB: Nuclear factor kappa-light-chain-enhancer of activated B cells
ROS: Reactive oxygen species
SIRT1: Sirtuin 1
TNFα: Tumour necrosis factor-alpha
TGF-β: Transforming growth factor beta
