# Supplementary figures and images for: Activation of Nrf2 Pathway Contributes to Neuroprotection by the Dietary Flavonoid Tiliroside
Source: Mol Neurobiol. 2018 Mar 5;55(10):8103–23. doi: 10.1007/s12035-018-0975-2 (PMC6132780; doi:10.1007/s12035-018-0975-2)

## Slide 1
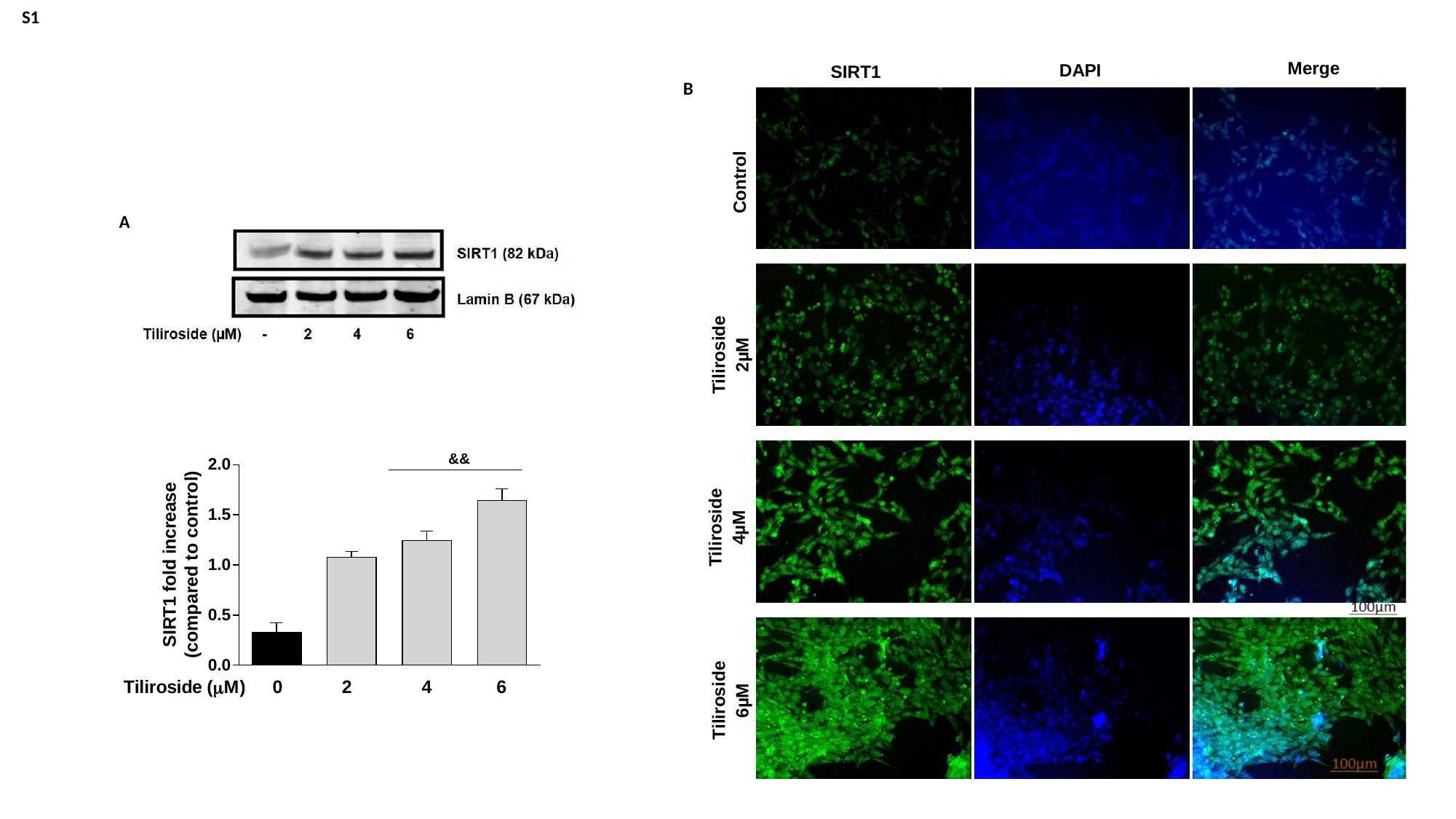

S1
B
A

Supplement: Supplementary file 1 — (S1): Tiliroside upregulated SIRT1 protein expressions in HT22 neuronal cells. (A) Neurons were incubated with tiliroside (2–6 μM) for 24 h. Later, nuclear extracts were collected and analysed for SIRT1 protein expression using western blot. (B) Immunofluorescence experiments were carried out to detect activation of SIRT1 by tiliroside in HT22 cells. Results reveal that very low levels of SIRT1 were observed in untreated cells while increasing concentrations of the compound induced SIRT1 activation and protein expression in HT22 neurons. All values are expressed as mean ± SEM for three independent experiments. Data were analysed using one-way ANOVA for multiple comparisons with post-hoc Student Newman-Keuls test. &p < 0.05, &&p < 0.01, &&&p < 0.001 compared with untreated control. (PPTX 3982 kb) [file 12035_2018_975_MOESM1_ESM.pptx]

## Slide 1
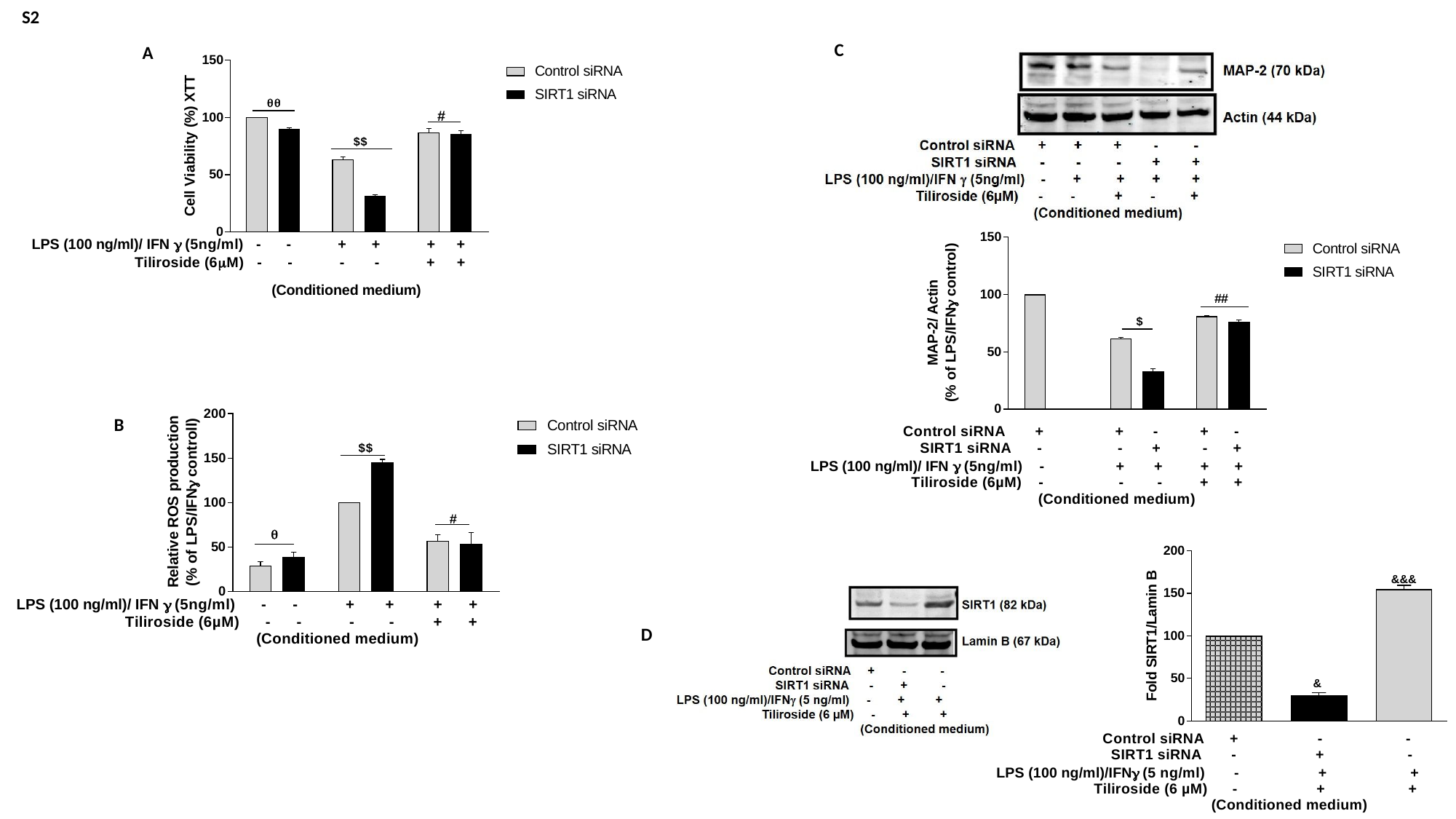

S2
C
A
B
D

Supplement: Supplementary file 2 — (S2): Neuroprotective activity of tiliroside is independent of SIRT1 protein activation in HT22 neurons. Cells were transfected with SIRT1 siRNA and control siRNA followed by incubation with conditioned medium containing LPS (100 ng/ml)/IFNγ (5 ng/ml) and tiliroside (6 μM) for 24 h. Thereafter, (A) XTT and (B) ROS generation assays were carried out. Results show that both cells that contained control and SIRT1 siRNA exhibited similar outcome. (C) Subsequently, cytoplasmic extracts were collected and subjected to western blotting to assess MAP2 expression. (D) Control siRNA and SIRT1 siRNA-transfected BV2 microglia, treated with tiliroside 6 μM for 24 h. Nuclear extracts were collected and assessed for SIRT1 expression using western blot. SIRT1 protein was significantly knocked down compared to control siRNA in HT22 neuronal cells. All values are expressed as mean ± SEM for at least three independent experiments. Data were analysed using one-way ANOVA for multiple comparisons with post-hoc Student Newman-Keuls test. θp < 0.05, θθp < 0.01, θθθp < 0.001 as compared within the groups of the untreated control. $p < 0.05, $$p < 0.01, $$$p < 0.001 as compared within the groups stimulated with LPS/IFNγ and #p < 0.05, ##p < 0.01, ###p < 0.001 as compared within the groups pre-treated with tiliroside (6 μM). p < 0.05, &&p < 0.01, &&&p < 0.001 compared with untreated control. (PPTX 286 kb) [file 12035_2018_975_MOESM2_ESM.pptx]
